# Validation of two immunoassays for oxytocin measurements in human saliva

**DOI:** 10.1371/journal.pone.0297539

**Published:** 2024-04-18

**Authors:** Marina López-Arjona, José Joaquín Cerón, Sandra V. Mateo, María Dolores Contreras-Aguilar, Silvia Martínez-Subiela

**Affiliations:** 1 Department of Animal and Food Science, Universitat Autònoma de Barcelona, Cerdanyola del Vallès, Barcelona, Spain; 2 Interdisciplinary Laboratory of Clinical Analysis of the University of Murcia (Interlab-UMU), Regional Campus of International Excellence ‘Campus Mare Nostrum’, University of Murcia, Murcia, Spain; 3 Molecular Inflammation Group, University Clinical Hospital Virgen de la Arrixaca, Biomedical Research Institute of Murcia (IMIB-Pascual Parrilla), Murcia, Spain; University of Pisa, ITALY

## Abstract

The objective of this research was to develop and validate two immunoassays for oxytocin measurement in human saliva, one using a monoclonal and the other a polyclonal antibody against oxytocin, whose affinity for oxytocin was tested by an antibody mapping epitope analysis. These assays were analytically validated and used to compare oxytocin concentrations with those obtained with a commercial kit before and after the extraction or reduction/alkylation (R/A) treatments to saliva samples. The assays were also used to evaluate changes in salivary oxytocin concentrations following a physical effort and an induced psychological stress, which have previously been described as situations that cause an increase in salivary oxytocin. Both assays showed to be precise and accurate in the validation studies, and the antibodies used showed a defined binding region in case of the monoclonal antibody, whereas the polyclonal antibody showed binding events through all the oxytocin sequence. Although the monoclonal and polyclonal assays showed a positive correlation, they give results in a different range of magnitude. Both assays showed significant increases in oxytocin concentrations when applied after the physical effort and the psychological stress. This study shows that a variability in the reported values of oxytocin can occur depending on the assay and indicates that the use of different types of antibodies can give a different range of values when measuring oxytocin in saliva.

## Introduction

Oxytocin is a peptide hormone synthesized in the hypothalamus. It reaches other brain areas via passive diffusion or collateral projections and is also being released into the blood for delivery to other tissues [1]. Physiological oxytocin functions are wide, involving reproduction and breastfeeding [2]. In addition, oxytocin is related to social behaviors such as affiliation between individuals [3, 4] or recognition [5], and it also has an anti-stress function [6, 7].

Oxytocin has been measured in different types of samples [8–11]. The use of saliva has the advantages of being non-invasive, easy to collect and non-stressful [12]. In addition, saliva has been demonstrated to be more sensitive to serum in some situations. For example, salivary oxytocin increased rapidly in mothers during direct interaction with the infant and/or when the mother was watching her own infant’s video, whereas no increase was observed in serum in these situations [13]. Commercially available enzyme-linked immunosorbent assay (ELISA) kits are usually used for oxytocin measurement in saliva in humans. In many cases, these assays need, prior to the analysis, an extraction [14] to remove some possible interferences, or lyophilization of sample to concentrate it [15], being procedures that increase their sensitivity. In addition, some kits need a reduction/alkylation (R/A) treatment, which liberates oxytocin from proteins, to measure the free oxytocin [16].

Recently, two different assays for oxytocin measurement in saliva, one using a monoclonal antibody and the second using a polyclonal antibody, have been validated in animal species [17–19] but not in humans. These assays use the AlphaLISA technology, which, compared with ELISA, is highly sensitive, requires low sample volume, is faster, and has no washing steps [20]. To the author`s knowledge, this technology has not been used to measure oxytocin in human saliva. In addition, there is no knowledge about the binding properties of the antibodies of these two different assays against oxytocin, as no mapping epitope study of each antibody has been performed.

One of the main open questions in the field of the saliva assays is the variability in the oxytocin concentrations detected by the different measurement methods. This can be due to the different form, or type of oxytocin that each assay measures (bound to protein, free form, and even oxytocin derived metabolites or oxytocin fragments, such as dimers or trimers) [21]. Also, it can be influenced by the selectivity and sensitivity of the antibody used or the binding interference of other molecules similar to oxytocin, like the vasopressin molecule [22, 23]. In addition, the sample processing, that can include an extraction procedure or a R/A, could have influence in oxytocin values [24]. Overall, it has been recently indicated that it is critical that researchers use standardized, valid, and reliable methods of oxytocin measurement and that these standardized methods to measure human oxytocin concentrations will improve the ability to interpret and understand human socioemotional processes and behavioural functioning [24].

This study aimed to validate two new assays for oxytocin measurement in human saliva, one of them using a monoclonal and the other using a polyclonal antibody. The assays were analytically validated according to recent published guidelines [24], and a mapping epitope study of each antibody against the oxytocin was also performed. Furthermore, the effect on the assays of two different sample processing methods, such as saliva extraction and a R/A, was evaluated. In addition, to evaluate the ability of these assays to detect physiological changes in oxytocin, these assays were applied to two different situations that have previously been described to produce an increase in oxytocin in saliva [9]: a physical effort (CrossFit trial) and induced psychological stress such as Trier Social Stress Test (TSST).

## Materials and methods

### Antibodies mapping epitopes

Epitope mapping of the monoclonal and polyclonal antibodies was conducted by Biosynth BV. (Lelystad, The Netherlands) following the protocol used by Gnanadesikan et al. [25]. Pepscan epitope mapping tests binding of an antibody to overlapping peptides synthesized from the target protein, thereby determining its epitope(s) and assessing its specificity. The goal was to map the epitopes of antibodies that recognize Oxytocin/Neurophysin I Propropeptide, using linear and conformational epitope mapping. A linear and conformational epitope mapping was employed, as well as a replacement analysis on the oxytocin sequence to identify key binding residues. To reconstruct epitopes of the target molecule a library of peptide-based mimics was synthesized using Fmoc-based solid-phase peptide synthesis. The binding of antibody to each of the synthesized peptides was tested in a Biosynth-based ELISA. This approach enables a comparison of the antibody’s affinity for different peptide sequences, which can shed light on the antibody’s specificity. Binding of antibodies to their antigens can be classified into different categories; binding can occur to continuous linear stretches of amino acids, to residues within a single strand that are arranged in specific structural conformations, and/or to residues that are present in parts of the protein or protein complex that are distant in sequence but adjacent in structure. To determine putative epitopes, several linear and conformational peptide mimic types were designed of different lengths. A monoclonal isotype control was used to confirm that binding peaks observed in the sample were specific and negative polyclonal control antibody was screened to evaluate potential non-specific binding.

### Immunoassays

#### Optimization of assays

Monoclonal assay. The production of the monoclonal anti-oxytocin antibody used in this assay has been previously described [20]. This is a direct competitive assay using AlphaLISA technology (PerkinElmer, MA, USA), in which acceptor beads are coated to the monoclonal antibody.

Polyclonal assay. The production of the polyclonal antibody used in this assay has been previously described [17]. This is an indirect competition assay using AlphaLISA technology, in which acceptor beads are coated to protein G (PerkinElmer, MA, USA).

For the optimization of the assays for human saliva samples, the biotinylated oxytocin, acceptor beads coated to the monoclonal antibody, polyclonal anti-oxytocin antibody, protein G acceptor beads and streptavidin donor beads were tested following a previously described procedure [20].

The saliva samples were diluted 1:2 with phosphate buffered saline (PBS) and added to the well where they were mixed with AlphaLISA universal buffer used for diluting the antibody.

#### Assays validation

The oxytocin-BSA (oxytocin conjugated to bovine serum albumin, Cusabio) was prepared for standard curve generation by diluting it in AlphaLISA Universal buffer. The concentrations used for the standard curve were as follows: 2400, 1200, 600, 300, 150, 75, 37.5 and 0 pg/mL in case of AlphaLISA monoclonal method and 200, 100, 50, 25, 12.5, 6.2, 3.1 and 0 ng/mL in case of AlphaLISA polyclonal method.

To assess the analytical validation of the assays, parameters recommended by Tabak et al. [24] were calculated. These parameters include imprecision, accuracy, and sensitivity, which comprises the limit of detection (LD) and the low limit of quantification (LLOQ). These parameters have been used in previous validations of oxytocin assays [24].

Imprecision was calculated as inter- and intra-assay variations and was expressed as coefficients of variation (CVs). Five replicates of each saliva sample (with low, medium, and high oxytocin concentrations) were analyzed at the same time to determine the intra-assay precision of the method. To determine the inter-assay precision, five aliquots of each saliva sample were stored in plastic vials at -80°C and were measured in duplicate over five different days using freshly prepared calibration curves.

Accuracy was assessed through linearity under dilution and recovery experiments. For linearity, two saliva samples were serially diluted with AlphaLISA Universal buffer, and the results were compared to the expected values using linear regression analysis. In the recovery experiment, saliva samples with different oxytocin concentrations were mixed with varying amounts of oxytocin-BSA. The percentage of recovery was calculated for each mixed sample by dividing the observed result by the expected result and multiplying by 100.

The LD was determined by measuring the AlphaLISA Universal buffer (blanks) in 12 replicates and calculating it as 2 times the standard deviation above the mean blank. The LLOQ was calculated by serially diluting saliva samples with different oxytocin concentrations with AlphaLISA Universal buffer and analyzing them five times in the same run. The lowest oxytocin concentration that could be measured with less than 20% imprecision was determined as the LLOQ.

### Participants

All participants were fully informed about the study, including the sampling times and objectives. Additional written informed consent was obtained from each individual participant, especially when identifying information was included in the article. The study was conducted with the approval of the Murcia University Ethics Committee (reference number: 1349/2016). Participants were not allowed to eat 1 h before the saliva collection and reported not having oral or systemic diseases. Participants were recruited between April and May 2020.

For the analytical validation and comparison between non-extracted, extracted samples and after the R/A procedure, the study included 12 saliva samples from adult healthy subjects, with an equal distribution of 6 females and 6 males. These samples were measured with the monoclonal and polyclonal method and compared with a commercially available ELISA kit from Cayman Chemical (Ann Arbor, MI, USA) used previously for oxytocin measurement in pig [17] and dog [26] saliva.

The samples used for the CrossFit trial and TSST were samples from previous studies that were stored at -80°C. The CrossFit trial involved 11 male participants who performed a CrossFit workout in a CrossFit training center (Murcia, Spain), according with previous study by Contreras-Aguilar et al. [27]. Saliva samples were collected at three time points: 5 minutes before the exercise (TBe), immediately after completing the exercise (T+0e), and 10 minutes after completion (T+10e). The entire experimental procedure took place between 18:00 and 20:00.

Saliva samples for the TSST were collected from 14 female university students, with an average age of 26.2 ± 6.26 years, and the TSST was conducted according to a previous study by Contreras-Aguilar et al. [27]. The participants performed the experimental procedure between 17:30 and 18:30. Each participant was isolated in a room where was asked to prepare for an interview for a job (5 min) and then confronted with two authoritatively and aloofly acting men investigators leading the 5-min interview session. The session finished by a 10-min arithmetic task in front of the investigators. These times were selected according to a previous study [9]. Saliva samples were obtained at three times: from each participant in the isolation room 5 min before the interview (TBs), just after the arithmetic task (that lasted 10 min) (T+0s), and 15 min later (T+15s).

All methods were carried out in accordance with relevant guidelines and regulations.

### Saliva samples

Participants rinsed their mouths carefully with tap water 1 hour before the collection. Saliva samples were collected by passive flow for 1 minute using standard micro-centrifuge polystyrene tubes with a volume of 5 mL, following the method described by Contreras-Aguilar et al. [27]. The samples were stored on ice until they arrived at the laboratory. Then, the samples were centrifuged (4500x g for 10 minutes at 4°C), and the supernatant was collected in Eppendorf tubes and stored at -80°C until analysis.

The R/A procedure was carried out following the protocol described by Brandtzaeg et al. [16], and 12 saliva samples from the CrossFit trial and TSST were treated with this procedure.

For the extraction procedure, aliquots of the same 12 saliva samples used for R/A procedure were used. The extraction procedure was performed through solid phase extraction following the method described by MacLean et al. [26]. All samples were diluted with 0.1% trifluoroacetic acid, centrifuged and saliva samples were run on separate Oasis PRiME cartridges (Waters Corporation). All samples were evaporated to dryness under a Speed vac Concentrator (Concentrator 5301, Eppendorf), and frozen at -80°C until assay.

Both R/A treated samples and extracted samples were measured with monoclonal and polyclonal AlphaLISA methods and Cayman kit, and compared with the same samples without extraction or R/A measured with the same methods.

### Statistical analysis

Descriptive statistics (intra-assay and inter-assay CVs) were calculated by routine analysis (Excel 2016, Microsoft). The Graph Pad Software Inc (GraphPad Prism, version 6 for Windows, Graph Pad Software Inc, San Diego, USA) was used for statistical analyses. Data obtained from different times of physical effort and TSST, as well as the values obtained in non-extracted, extracted and R/A samples, were evaluated for normality of distribution, using the Shapiro-Wilk test, resulting in a nonparametric distribution with monoclonal and polyclonal method. Values were log-transformed, and a one-way Analysis of Variance (ANOVA) followed by Tukey’s test was used to compare oxytocin concentrations in non-extracted, extracted and R/A samples within each method. Pearson correlation coefficients were calculated to evaluate the correlation between non-extracted, extracted and R/A samples within each method, as well as between monoclonal and polyclonal method in physical effort and TSST samples without extraction. The results of oxytocin concentrations obtained in physical effort and TSST were log-transformed, and a one-way Repeated Measures ANOVA followed by Tukey’s test was used, to compare values obtained in participants at different times and to determine if there were any significant differences. For comparison between oxytocin values of monoclonal and polyclonal method with physical effort and TSST, a Regression plot was used in which the X-axis shows the results obtained with the monoclonal antibody and the Y-axis those obtained with the polyclonal one (these results were expressed in ng/mL). Results were reported as mean and standard deviation (SD) (in the text: mean ± SD) and lines (in Figures), and a P < 0.05 was considered significant.

## Results

### Antibody mapping epitope

The analysis results established tentative epitopes for the two antibodies used in the assays of this study. The monoclonal antibody showed a defined binding region, whereas the polyclonal sample showed binding events through all the sequence.

For the monoclonal antibody, clear binding peaks were observed with little background. The antibody could be used at relatively low concentrations. Binding peaks observed in the sample were specific when a monoclonal isotype control was used. The epitope mapping results identified binders within the sequence oxytocin/neurophysin I prepropeptide, specifically within sequence SACYIQNCPLGGKRAAPDL. A replacement analysis was done on the oxytocin sequence, and identified nine key residues for binding (CYIQNCPLG). The most pronounced decrease in binding was observed for mutations in glutamine (Q), glutamate (N), and proline (P), which suggests these are critical binding residues in the epitope. The flanking isoleucine (I) and leucine (L) were affected as well but not as prominently.

For the polyclonal antibody, multiple binding peaks were found with linear and conformational mapping. Binding peaks observed in the sample were specific when a polyclonal control was used. The replacement analysis on the oxytocin sequence also did not show clear distinct key residues that were affected by mutation. The polyclonal shows many small binding regions, possibly due to multiple clones raised against a small antigen.

### Optimizations and validation of the assays

**Fig 1:**
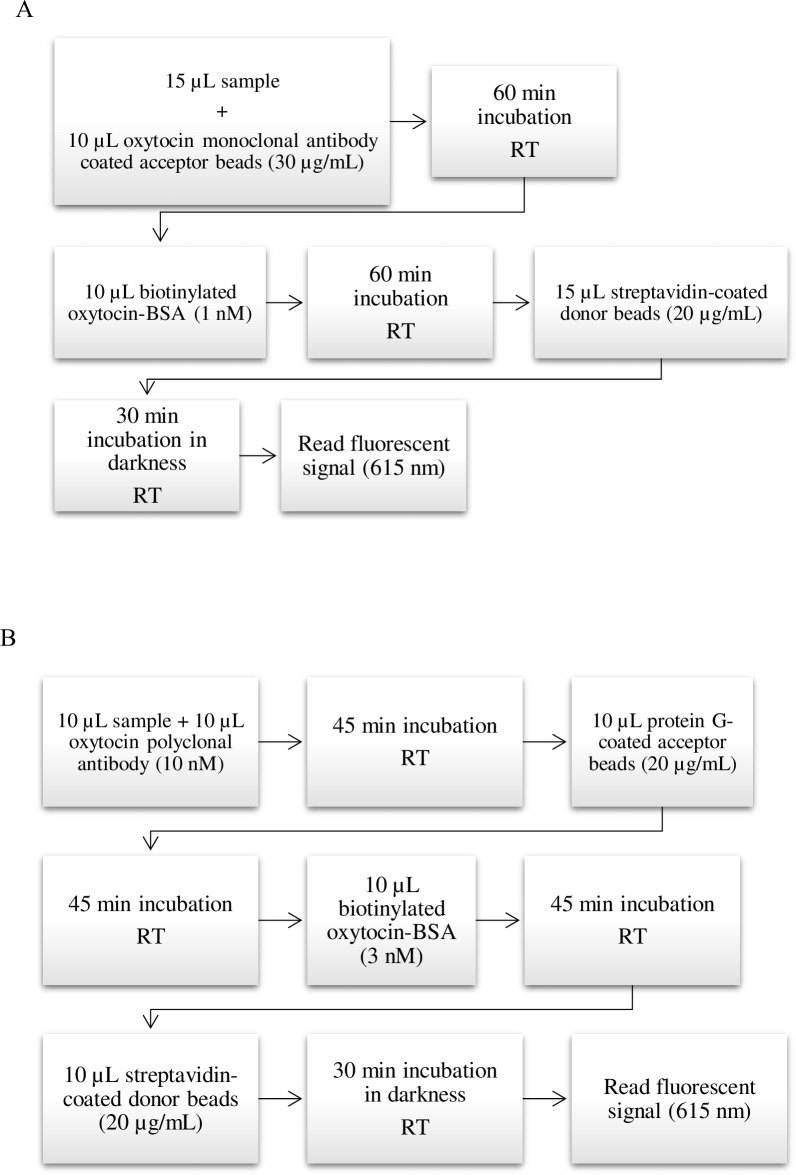
AlphaLISA protocol with the monoclonal (A) and polyclonal (B) antibody.

The assays were performed in 96 well plates (PerkinElmer, Inc., USA) with a total volume of 50 μL per well. The resulted protocols are shown in Fig 1.

### Precision, intra and inter-assay CV, dilution linearity and recovery

Monoclonal method. The intra-assay and inter-assay CVs were 2.2–12.7% and 8.0–16.0%, respectively. The results of recovery were between 81% and 115%. The assay LD was 54.8 pg/mL and the LLOQ was 72.5 pg/mL. The linear regression equations resulted in a correlation coefficient between 0.96 and 0.97.

Polyclonal method. The intra-assay and inter-assay CVs were 3.8–11.9% and 8.4–14.5%, respectively. The results of recovery were between 91% and 120%. The assay LD was 0.5 ng/mL and the LLOQ was 11.5 ng/mL. The linear regression equations resulted in a correlation coefficient ranged between 0.96 and 0.99.

The recovery results are shown in Table 1.

**Table 1:** Recovery results of monoclonal and polyclonal assays.

Analyte	Assay	Sample oxytocin concentration	Oxytocin amount added	Expected values	Resulted values	Recovery rate (%)
Oxytocin-BSA	Monoclonal (pg/mL)	1807.0	0	1807	1807	100
			187.5	997.2	895.5	89.8
			375	1091.0	941.4	86.3
			750	1278.5	1396.5	109.3
			1500	1653.5	1343	81.2
		439.6	0	439.6	439.6	100
			187.5	313.6	316.2	100.8
			375	407.3	447.8	109.9
			750	594.8	543.0	91.3
			1500	969.8	1113.0	114.8
	Polyclonal (ng/mL)	126.8	0	126.8	126.8	100
			7.5	67.2	78.5	116.8
			15	70.9	69.3	97.7
			30	78.4	69.2	88.3
			60	93.4	107.9	115.5
		32.1	0	32.1	32.1	100
			7.5	19.8	16.8	84.8
			15	23.5	27.4	116.6
			30	31.1	30.2	97.1
			60	46.1	42.1	91.3

### Effects of extraction and R/A treatment

**Fig 2:**
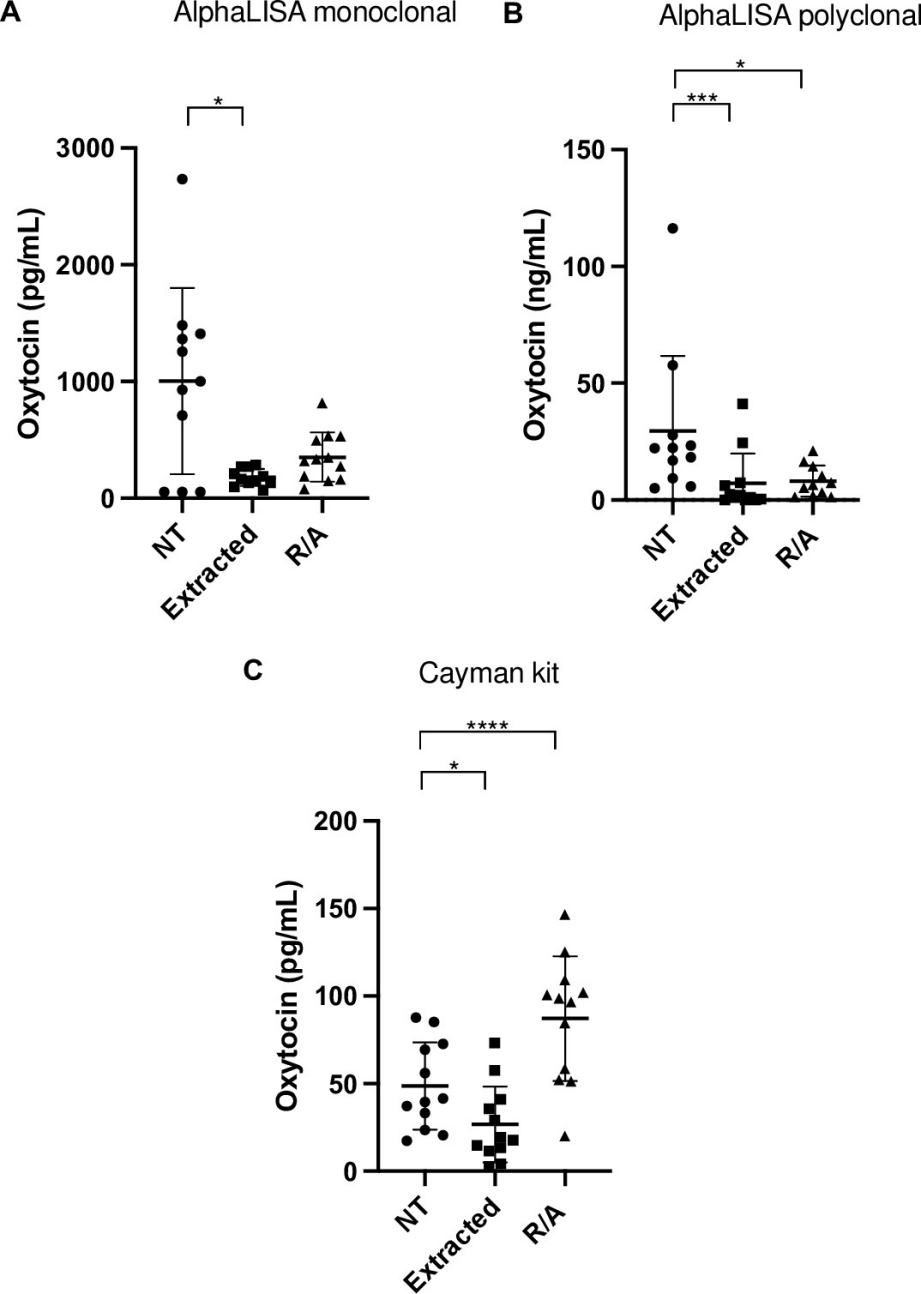
Oxytocin concentrations in human saliva before (non-extracted) and after extraction (Extracted) and R/A treatment (R/A) with alphaLISA monoclonal method (A), alphaLISA polyclonal method (B), and kit Cayman (C). Asterisks indicate significant differences (*P < 0.05; ***P < 0.001; ****P < 0.0001). The graphics show means means ± Standard deviation and individual values (circles, squares and triangles).

The oxytocin concentrations in saliva samples without treatment, with extraction and R/A procedure are shown in Fig 2. Significant changes in oxytocin concentrations were obtained with monoclonal method (F_2,31_ = 4.09, P = 0.027), polyclonal method (F_2,31_ = 9.07, P = 0.0008) and Cayman kit (F_2,33_ = 12.33, P = 0.0001) between treatments. The correlations between non-extracted and extracted with each method, and non-extracted and R/A procedure with each method are shown in Tables 2 and 3, respectively.

**Table 2:** Pearson correlation coefficients between different methods without (non-extracted) and with extraction (extracted).

	AlphaLISA monoclonal non-extracted	AlphaLISA monoclonal extracted	AlphaLISA polyclonal non-extracted	AlphaLISA polyclonal extracted	Cayman kit non-extracted	Cayman kit extracted
**AlphaLISA monoclonal non-extracted**		0.706* (0.022)	0.702** (0.001)	0.088 (0.796)	0.462 (0.210)	0.345 (0.299)
**AlphaLISA monoclonal extracted**			0.679* (0.021)	0.034 (0.921)	0.828** (0.001)	0.167 (0.623)
**AlphaLISA polyclonal non-extracted**				-0.122 (0.704)	0.549 (0.125)	0.400 (0.197)
**AlphaLISA polyclonal extracted**					-0.022 (0.955)	-0.577 (0.050)
**Cayman kit non-extracted**						-0.045 (0.908)
**Cayman kit extracted**						

Asterisks indicate statistical significance (

* P < 0.05

** P < 0.01) and P-values are indicated in brackets.

**Table 3:** Pearson correlation coefficients between different methods without (no R/A) and with R/A treatment (R/A).

	AlphaLISA monoclonal no R/A	AlphaLISA monoclonal R/A	AlphaLISA polyclonal no R/A	AlphaLISA polyclonal R/A	Cayman kit no R/A	Cayman kit R/A
**AlphaLISA monoclonal no R/A**		0.574* (0.043)	0.702** (0.001)	0.256 (0.290)	0.462 (0.210)	0.562 (0.091)
**AlphaLISA monoclonal R/A**			0.307 (0.111)	0.443 (0.094)	0.764* (0.027)	0.608 (0.062)
**AlphaLISA polyclonal no R/A**				0.201 (0.370)	0.549 (0.125)	0.530 (0.093)
**AlphaLISA polyclonal R/A**					0.566 (0.112)	0.466 (0.148)
**Cayman kit no R/A**						0.308 (0.458)
**Cayman kit R/A**						

Asterisks indicate statistical significance (

* P < 0.05

** P < 0.01) and P-values are indicated in brackets.

When AlphaLISA monoclonal method was used, the values obtained in the samples without extraction (478.5 ± 365.0 pg/mL) were significantly higher than those with extraction (178.8 ± 72.9 pg/mL) (P = 0.0200), and a significant positive correlation were found between extracted and non-extracted samples (r = 0.706, P = 0.022). The values obtained in the samples without extraction were higher but did not show significant differences when compared with the values obtained with the R/A procedure (377.1 ± 201.3 pg/mL) (P = 0.3077). No significant correlation was found between non-extracted samples and R/A (r = 0.574, P = 0.042). Within the samples without treatment, three of them fell below the LD of the assay.

When AlphaLISA polyclonal method was used, the values obtained in the samples without extraction (29.6 ± 32.1 ng/mL) were significantly higher than those with extraction (7.2 ± 12.7 ng/mL) (P = 0.0006), and no significant correlation were found between extracted and non-extracted samples (r = -0.122, P = 0.705). R/A procedure produced a significant decrease in oxytocin values (8.1 ± 6.7 ng/mL) compared to non-extracted samples (P = 0.0364). A significant positive correlation was found between non-extracted samples and R/A (r = 0.201, P = 0.370). Within the samples with extraction, three of them fell below the LD of the assay.

When Cayman kit was used, the values obtained in the samples without extraction (48.7 ± 24.9 pg/mL) were significantly higher than values after extraction (26.7 ± 21.7 pg/mL) (P = 0.0222), and no significant correlation were found between extracted and non-extracted samples (r = -0.045, P = 0.908). The R/A procedure showed a significant increase in oxytocin values (87.2 ± 35.6 pg/mL) compared to non-extracted samples (P < 0.0001). No significant correlation was found between non-extracted samples and R/A (r = 0.308, P = 0.458). Withing the samples without treatment, two of them fell below the LD.

### Physical effort

Significant changes in oxytocin concentrations were obtained with monoclonal method (F_2,20_ = 4.55, P = 0.023) and polyclonal method (F_2,19_ = 4.73, P = 0.022) between times.

When the AlphaLISA monoclonal method was applied, the participants in the CrossFit WOD model showed a significant increase in oxytocin at T+0e (5265.0 ± 4476.0 pg/mL) compared with TBe (1479.0 ± 1404.0 pg/mL) (P = 0.0397) and T+10e (1923.0 ± 2343.0 pg/mL) (P = 0.0446).

When the polyclonal method was applied, a significant increase in oxytocin was detected at T+0e (134.7 ± 104.5 ng/mL) compared with TBe (58.8 ± 38.1 ng/mL) (P = 0.0400) and T+10e (71.9 ± 62.7 ng/mL) (P = 0.0405).

### TSST

No significant changes in oxytocin concentrations were obtained with monoclonal method (F_2,24_ = 3.31, P = 0.054) and polyclonal method (F_2,23_ = 3.24, P = 0.058) between times. The results obtained with alphaLISA monoclonal method in saliva samples of the TSST model showed a significant increase in oxytocin at T+0s (1455.0 ± 1236.0 pg/mL) compared with TBs (758.5 ± 975.1 pg/mL) (P = 0.0452) but did not show significant differences with T+15s (1061.0 ± 949.5 pg/mL) (P = 0.6593).

The results obtained with alphaLISA polyclonal method in saliva samples of the TSST model showed a significant increase at T+0s (82.6 ± 58.8 ng/mL) compared with T+15s (48.3 ± 39.3 ng/mL) (P = 0.0471) but did not show significant differences with TBs (68.1 ± 61.1 ng/mL) (P = 0.5846).

**Fig 3:**
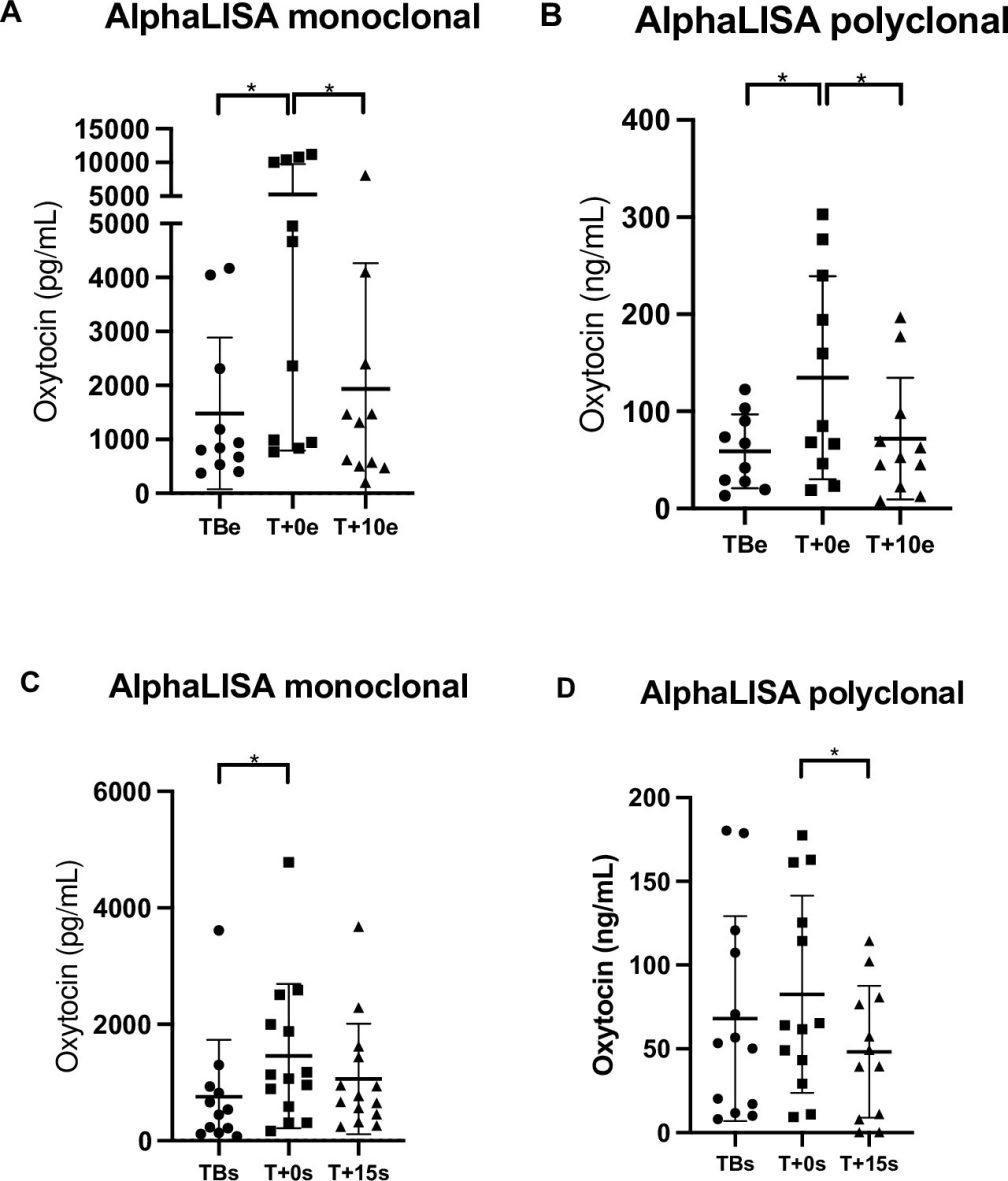
Oxytocin concentrations in saliva samples of the participants during the physical effort and the psychological stress. The oxytocin concentrations were measured 5 min before the exercise (TBe), after completion of the exercise (T+0e), and 10 min after the exercise (T+10e) with AlphaLISA monoclonal (A) and polyclonal (B) method, and 5 min before the interview (TBs), just after the arithmetic task (T+0s), and 15 min later (T+15s) with AlphaLISA monoclonal (C) and polyclonal (D) method. The graphics show means ± Standard deviation and individual values (circles, squares, and triangles). Asterisks indicate significant differences between different times (*P < 0.05).

**Fig 4:**
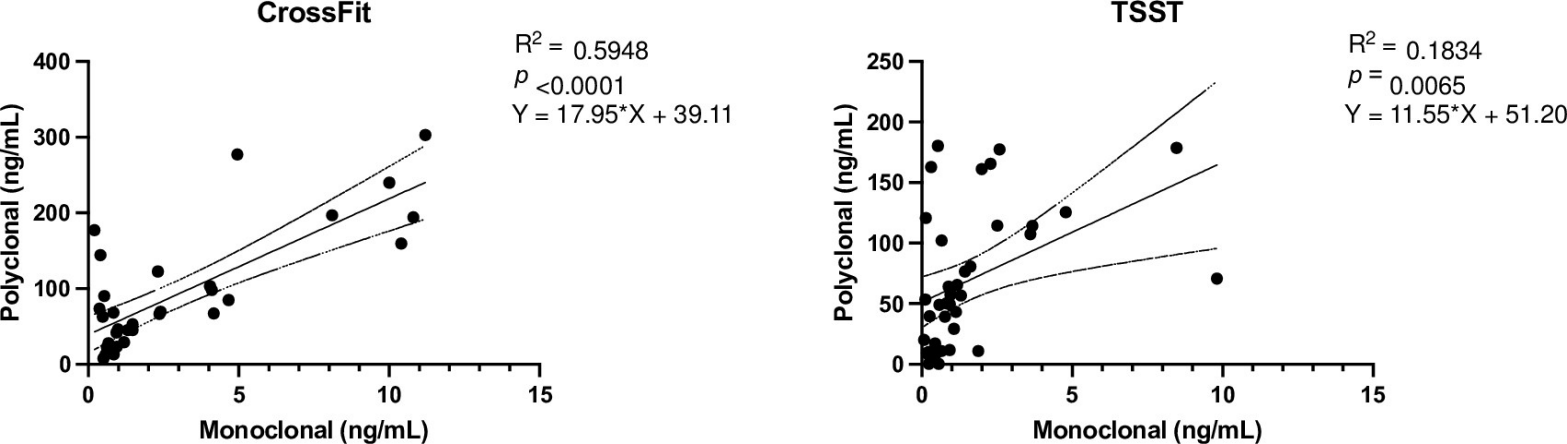
Linear regression between salivary oxytocin concentrations with monoclonal vs. **polyclonal method in CrossFit and TSST model.** Circles show oxytocin concentrations. The continuous line shows linear regression, and the dotted lines show the 95% confidence interval. R^2^: Coefficient of linear regression.

The results of oxytocin concentrations in both models are shown in Fig 3 and the regression plots between both methods in Fig 4.

Regression assays showed slopes significantly far from zero and determination coefficients R2 of 0.59 for the CrossFit trial and 0.18 for the TSST trial.

## Discussion

In this report, two new methods for measuring oxytocin in human saliva were developed and validated, one using a monoclonal antibody and one using a polyclonal antibody. These antibodies have been previously used for oxytocin measurement in the saliva of pigs and other animal species such as dogs and cows [17, 19, 20, 28]. The validation carried out in this study for the new methods developed to measure oxytocin concentrations in human saliva followed the technical procedures previously recommended for the validation of oxytocin assays (antibody selectivity and specificity, assay sensitivity, inter and intra-assay CV, dilution linearity, precision, and recovery) [24].

In both assays, oxytocin conjugated to BSA (bovine serum albumin) was used to generate the standard curve. BSA not only contributes to the stability of oxytocin but also aids in stabilizing short-sized molecules in general [29]. The practice of linking molecules to BSA has been widely employed as a standard calibration approach in various immunoassays [30]. Importantly, BSA did not exhibit any reactivity with the antibodies used in the study when tested at different concentrations as a sample. Thus, it did not interfere with the reaction, ensuring the accuracy of the measurements.

The described methods in this study were characterized by their precision and high linearity, even after serial dilutions. These methods offered several advantages compared to many commercially available ELISA kits, including the use of lower sample volumes and the absence of washing steps, which simplified the procedure. Furthermore, the sensitivity of these methods was sufficient to detect oxytocin in saliva without the need for lyophilization, which is necessary for some less sensitive assays [15, 29].

The assays validated in our study gave oxytocin values in the saliva of different orders of magnitude: pg/mL in the case of the monoclonal assay and ng/mL in the case of the polyclonal assay. These differences in magnitudes were also previously reported when both assays were used to measure a synthetic oxytocin solution [17]. The reasons for the different magnitude of the obtained values and the behavior of each assay during sample extraction or the R/A procedure could be various. (1) One would be the different selectivity or sensitivity of the two antibodies for the oxytocin molecule that was confirmed in the epitope study of the antibodies and it has also been previously reported with commercially immunoassays having oxytocin antibodies that varied in the epitopes recognized [31]. (2) Differences in recognition of different oxytocin forms or metabolites. Previous studies have reported that the oxytocin molecule can appear bound to other proteins or lipids, and also, there are different forms or fragments of oxytocin that can be present in multiple dynamic states. In this line, differences in selectivity and sensitivity of the antibody used or the binding interference of other molecules similar to oxytocin or immune reactive metabolites have been described as causes of variation of the results of different assays [22, 31]. (3) Finally, the antibodies could have a different degree of interference by molecules such as plasma proteins other than oxytocin itself that can produce a high baseline value [23]. It could also be the reason why in previous reports, different oxytocin values and ranges are obtained when different assays are used, such as mass spectrometry [32], ELISA [14] or radioimmunoassay (RIA) [9].

The epitope mapping of our study showed different results according to the type of antibody used. The fact that in case of the monoclonal antibody the peaks were relative clear, and several binding peaks were found with linear and conformational mapping, while with the polyclonal multiple poor definition of peaks were found, could indicate a higher specify reaction between monoclonal antibody and the oxytocin molecule. The reasons of the finding obtained with the polyclonal antibody, could be due to the fact several antibody clones exist that can bind to multiple parts of the antigen. This is in concordance with previous studies that indicate polyclonal antibodies may exhibit unwanted reactions, including cross-reactivity [33]. López-Arjona et al. [17] also concluded that polyclonal antibody against oxytocin could bind to other oxytocin metabolites or forms because the concentrations were higher than with the monoclonal antibody using the AlphaLISA technology. Interestingly, the key residues of the epitope recognised by the monoclonal antibody are residues 3–9 of the oxytocin molecule. This may explain the ability of our monoclonal antibody to detect oxytocin, as well as the lack of cross-reactivity of this antibody with vasopressin that has been previously reported [20], since this nonapeptide differs from oxytocin at residues three and eight.

It could be hypothesized that the polyclonal assay could have more affinity by the oxytocin bound to proteins since it showed a significant decrease with the R/A procedure. In addition, the higher values found in comparison with the monoclonal assay could be influenced by its ability to detect other oxytocin forms or metabolites; although also possibly would have more interferences with other compounds than the monoclonal antibody. Similar results were obtained in pigs and dogs [17, 18]. The monoclonal assay showed a decrease in the values of less magnitude and non-significant after R/A treatment, and this could be possible because it could have more affinity for the oxytocin that can be liberated by the R/A procedure. In addition, regarding the commercial kit used in our study, it would need the R/A procedure for wider recognition of the oxytocin or oxytocin-like molecule as has been previously reported in human plasma [16].

The different behavior of the assays during the extraction process suggests that they might be measuring different components or forms of oxytocin. In the case of the monoclonal assay, a significant correlation was observed between the values obtained before and after the extraction, which is consistent with previous findings in the saliva of pigs [20] and dogs [26] and that could indicate the not need of extraction with this assay. On the other hand, the polyclonal assay did not show any correlation between extracted and non-extracted samples, which is similar to findings reported in a different assay using a polyclonal antibody against oxytocin in human serum [34]. This disparity in behavior could be attributed to the potential loss of oxytocin bound to proteins or other forms of oxytocin, as well as other molecules that might be eliminated during the extraction procedure. Therefore, care should be taken in the case of extraction procedure in the case of the polyclonal assay since it can produce a significant variation in the results. In any case, both the monoclonal and polyclonal assays of our study showed high linearity in samples serially diluted before the extraction procedure, so it would not be needed to perform an extraction with these assays [35].

The mean value of oxytocin obtained with the monoclonal assay in the validation study without extraction (478 pg/mL) was higher than other values reported in the literature and also than the values we obtained with the Cayman`s kit (48.7 pg/mL). However, these values in a similar range to those reported by other authors in human saliva samples without extraction such as Nishizato et al. [36] with values up to 350 pg/mL and Moussa et al. [37] with values up to 360 pg/mL. Even in plasma samples values of 4 digits have been reported such as Lefevre et al. [11] with mean values of 1487.2 pg/mL and Glenk et al. [38], with values up to 1400 pg/mL. This will indicate a variability in the values obtained for oxytocin in saliva depending on the assay used as has been previously described [21].

The changes in salivary oxytocin concentrations found in this study in the CrossFit test agree with previous studies that measured oxytocin in saliva after physical efforts [9]. Although these authors used a different assay for the measurement of oxytocin, the magnitude of increase in oxytocin concentrations after the physical effort reported by de Jong et al. [9] was approximately 2.5-fold, while in our study, the increase was 3.5 and 2.2 fold with the monoclonal and polyclonal method, respectively.

In addition, the changes found in oxytocin in saliva in our study after the laboratory social stressor (TSST) agree with previous reports [9]. In this case, the magnitude of increase in oxytocin concentrations after the TSST reported by de Jong et al. [9] was approximately 2.0-fold, while the increase was 1.9 and 1.2-fold with the monoclonal and polyclonal method, respectively.

In other previous studies where TSST were performed, the increased magnitude of oxytocin in saliva was approximately 1.3, 1.6 and 1.1-fold [39–41], although in some of them the post-test time was after 5 min [42]. We found high inter-individual variability in the oxytocin values with both assays that lead to high standard deviations specially in measures made after the test as previously described [38, 41, 42], being this variability higher in the measurements after the task [29] as occur in the present study.

Regression plots showed that a significant linear relationship exists between the oxytocin results obtained with both methods in the two situations (CrossFit and TSST) used to evaluate the oxytocin response in our study. In spite of this, this relationship seemed to be dependent of the physiological situation, since the results observed with the monoclonal method predicts over 50% of those proportioned by the polyclonal one in the CrossFit trial, whereas this percentage decreases to 18% in the TSST trial. This could be due to the fact that the polyclonal antibody detects also bound oxytocin and its metabolites, which could vary depending on the trial. Also, based on the results of the two trials of our study, using the monoclonal assay to evaluate oxytocin changes in these situations could be recommended; since polyclonal appears to be less sensitive and/or less specific, maybe because it can bind to other molecules than oxytocin, and the large molecules to which oxytocin is bound can vary from person to person and in different physiological states [24]. Different sensitivity of the monoclonal and polyclonal assays has been previously reported in dogs [18].

For the interpretation of the two assays used in our study, based on the current knowledge, it can be indicated that the monoclonal assay would detect the free and bound oxytocin, whereas the polyclonal have more affinity for oxytocin bound to proteins (it showed a significant decrease with the R/A procedure that liberates oxytocin from proteins) and possibly for oxytocin metabolites (as stated by the epitope mapping study). The relation between the monoclonal and polyclonal assays and their responses can vary depending on the stressor stimulus, as it has occurred in our report. Further studies in different biological situations should be performed to evaluate how monoclonal and polyclonal assays behave in these situations and therefore gain knowledge about their interpretation.

As limitations of this study, the oxytocin concentrations measured with our methods have not been compared with mass spectrometry, although this technique can not detect bound oxytocin or related molecules [21], and currently, there is no gold standard for oxytocin measurements. In this line, it would be of interest to evaluate in more detail and define which components of oxytocin are detected by each method. Additionally, other models should be studied to evaluate the ability of the methods to detect changes in oxytocin in different physiological or stressful situations. From a more general point of view, it will also be of interest to clarify how oxytocin reaches saliva.

## Conclusion

This report describes two assays for oxytocin measurements in saliva that do not need lyophilization and/or extraction and that can detect changes in oxytocin in situations of physical effort or psychological stress. Each of these assays provided a different range of values and behaved differently in the experimental situations of our study, probably because they have a distinct affinity for oxytocin or detect diverse oxytocin forms. In addition the polyclonal assay can have interferences due to cross-reactivity with other plasma proteins not related to oxytocin, that could lead to high basal values.
